# Inequalities in Healthcare Access, Experience and Outcomes in Adults With Inflammatory Bowel Disease: A Scoping Review

**DOI:** 10.1093/ibd/izae077

**Published:** 2024-04-11

**Authors:** Rachel L Hawkins, Maryam Zia, Daniel Hind, Alan J Lobo

**Affiliations:** Sheffield Centre for Health and Related Research, School of Medicine and Population Health, University of Sheffield, Sheffield, United Kingdom; Sheffield Centre for Health and Related Research, School of Medicine and Population Health, University of Sheffield, Sheffield, United Kingdom; Sheffield Centre for Health and Related Research, School of Medicine and Population Health, University of Sheffield, Sheffield, United Kingdom; Sheffield Inflammatory Bowel Disease Centre, Sheffield Teaching Hospitals NHS Foundation Trust, Sheffield, Sheffield, United Kingdom

**Keywords:** inflammatory bowel disease, health inequalities, scoping review

## Abstract

**Background:**

Inflammatory bowel diseases (IBDs) are incurable diseases that require lifelong access to health services. Accumulating evidence of inequalities in health care access, experience, and outcomes for individuals with IBD is apparent. This review aimed to describe the inequalities in healthcare access, experiences, and outcomes of care for adults with IBD, to identify research gaps, and to identify future research priorities in this area.

**Methods:**

A scoping review was conducted to retrieve quantitative, qualitative, and mixed methods evidence from 3 databases (EMBASE, Medline, and CINAHL) published between January 1, 2000, and September 27, 2023.

**Results:**

Fifty-one studies met the criteria for inclusion. The majority (42 of 51) focused on IBD health outcomes, followed by healthcare access (24 of 51). Significantly fewer investigated patient experiences of IBD healthcare (8 of 51). Most available studies reported on race/ethnic disparities of healthcare (33 of 51), followed by inequalities driven by socioeconomic differences (12 of 51), rurality (7 of 51), gender and sex (3 of 51), age (2 of 51), culture (2 of 51), literacy (1 of 51), and sexuality (1 of 51). Inflammatory bowel disease patients from Black, Asian, and Hispanic ethnic groups had significantly poorer health outcomes. A lack of research was found in the sexual and gender minority community (1 of 51). No research was found to investigate inequalities in IBD patients with learning disabilities or autism.

**Conclusions:**

Further research, particularly utilizing qualitative methods, is needed to understand health experiences of underserved patient populations with IBD. Cultural humility in IBD care is required to better serve individuals with IBD of Black and Asian race/ethnicity. The lack of research amongst sexual and gender minority groups with IBD, and with learning disabilities, poses a risk of creating inequalities within inequalities.

**Key Messages**

**What is already known?**

Disparities in healthcare access, experience, and outcomes for individuals with inflammatory bowel disease (IBD) are highly prevalent.

**What is new here?**

This is the first review to synthesize IBD inequalities across issues of access, experience, and outcomes of care across multiple social drivers of inequality. We identified significant deficits in knowledge surrounding patient experiences of care across underserved groups, and in particular, IBD populations including the sexual and gender minority community and people with learning disabilities.

**How can this study help patient care?**

Understanding of the existing inequalities amongst vulnerable groups with IBD is important for clinicians and in the organization of IBD services to equitably care for these individuals.

## Introduction

### Background

Inflammatory bowel diseases (IBDs) includes Crohn’s disease (CD), ulcerative colitis (UC), and an “unclassified” IBD-U.^1^ Due to the chronic and unpredictable nature of the diseases, people with IBD require lifelong interactions with healthcare services.^2^ Upon an IBD diagnosis, an individual may experience frequent visits to outpatient care for symptom management and require more than 1 acute inpatient care for treatment during a flare, including surgical interventions.^2^ However, research shows that unequal access, experiences, and outcomes of healthcare exist for patients with IBD.^3,4^ Diverse groups are also underrepresented in the trials of pharmacological agents that inform clinical practice.^5^

Health inequalities are unfair and avoidable differences in the health and wellbeing of people that arise due to unequal distribution and access to health and social care.^6,7^ Such inequalities occur due to unequal conditions of daily life and the fundamental drivers of inequity: money, power, and resource.^8^ Health inequity refers to an injustice and unfairness that is perpetuated, and it is these inequities that cause inequalities in health.^9^ Vulnerable populations with IBD have greater rates of avoidable emergency admissions in IBD,^10^ implying healthcare inequity. A previous review of patient experiences of chronic bowel conditions found cultural, religious, language, and health literacy barriers prominently influenced the health inequity experienced by patients from Black, Asian, and other ethnic communities.^11^

Much of the disparities in access and experience of IBD care are attributed to poor access to specialist care and gastroenterologists.^3,4^ Social determinants of health, “the conditions in which people are born, grow, live, work and age,”^8^ are found to exacerbate existing inequalities in access and experience of care and subsequent health outcomes for patients with IBD.^4,12,13^ However, whilst multiple disparities are known in the care of IBD patients, there appears no existing literature review to synthesize knowledge across social determinants to identify research gaps and priority areas.

Traditionally, reducing health inequalities has drawn attention to individuals, local authorities, public health policy, and social care.^14^ Health systems arguably play a vital role in tackling social determinants of health to reduce healthcare-driven inequalities impacting patient access, experience, and health outcomes.^15,16^ There is a breadth of disadvantage and difference between healthcare and health outcome inequalities, and as such, health systems are required to advocate and work together to tackle health inequalities.^14^

There is no existing systematic review of the literature that maps existing inequalities in IBD access, experience, and outcomes across various social drivers of inequality. Previous reviews have been outcomes focused^17^ and not on synthesizing patient access and/or experience. The reason for focusing on access, experience and outcome consequences of inequality were determined from UK initiatives from NHS England in reducing healthcare inequalities.^16^

This scoping review synthesized the existing empirical evidence on the inequalities relating to IBD patient access, experience, and outcomes of healthcare across a broad range of social determinants of inequality (ethnicity/race, sexual and gender minority, age, sex and gender, rurality, socioeconomic factors, and culture) to help in service design and provision and with the aim of identifying research gaps and priorities to guide future research in this area.

## Methods

This review was conducted according to PRISMA-ScR guidance^18^ and the 5 stages framework for conducting scoping reviews.^19^

### Eligibility Criteria

A set of inclusion and exclusion criteria was applied. To do this, the “population, concept, and context” (PCC) mechanism was used.^20^ Peer-reviewed, primary research articles were eligible. We included quantitative and mixed-methods designs that were accessible in English. Conference abstracts, commentaries, and systematic reviews were ineligible for this review. We did, however, manually scan reference lists of relevant systematic reviews to identify relevant primary research articles for inclusion. The PCC criteria stated:

- Population: adults (>16 years) with any diagnosis of IBD. We excluded non-IBD populations and pediatric IBD populations.
- Concept: Included articles investigated a broad range of health inequalities related to access experiences and outcomes of IBD healthcare. Studies that were focused on IBD outcomes such as quality of life or disability without reference to access, experience, or outcomes of care were excluded. We also excluded basic science studies and prognostic factor studies. Eligible articles in this review included but were not limited to social determinants of:
  ○ Race and ethnicity (as reported by investigators) are dynamic social constructs which are shaped by cultural, geographic, and sociopolitical forces^21^
  ○ Cultural factors: Defined as beliefs, practices, and behaviors that are defined by customs, habits, language, and geography that groups of individuals share^22^
  ○ Socioeconomic factors (including deprivation, employment status, education, and housing status)
  ○ Gender or sex (as investigated and reported by investigators)
  ○ Geographic location and rurality
  ○ Sexual and gender minority communities: Including inequalities within the lesbian, gay, bisexual, transgender, queer, intersex, asexual, and other groups (LGBTQIA+)
- Context: We included articles from all countries focused on access, experience of outcomes of healthcare received by patients with IBD. If articles were not relevant to the healthcare received by patients with IBD, these were excluded. These included, for example, public health interventions and health policy analyses.

### Information Sources

A search was conducted in EMBASE via Ovid, Medline via Ovid, and CINAHL from January 1, 2000, to September 27, 2023. Initial scoping searches were performed to which these databases were identifying eligible research articles already known to the review team. Citation searching of relevant articles, including excluded systematic reviews, identified in the search was also used.

### Search Strategy

The search strategy was iteratively developed by 3 researchers (M.Z., R.H., and D.H.) using terms identified from previous systematic reviews on health inequalities and iteratively developed by piloting the search strategy until a satisfactory number of articles was produced. One researcher (M.Z.) conducted the initial search and applied a set of search criteria to all databases. The search strategy applied Boolean searching and combined MeSH terms for IBD (eg, inflammatory bowel disease/crohn disease/ulcerative colitis) and MeSH and free-text terms of healthcare access, experience, and/or outcomes (eg, healthcare disparity/healthcare access/) and social inequality terms (eg, minority group/rural healthcare/ethnic group/). A second researcher (R.H.) ran the search using the same search criteria and added LGBTQIA+ search terms to expand the scope of the review (eg, sexual minority/“LGBT people”/exp). A copy of the full search strategy for each database can be found in the appendix.

### Study Selection

**Figure 1.**
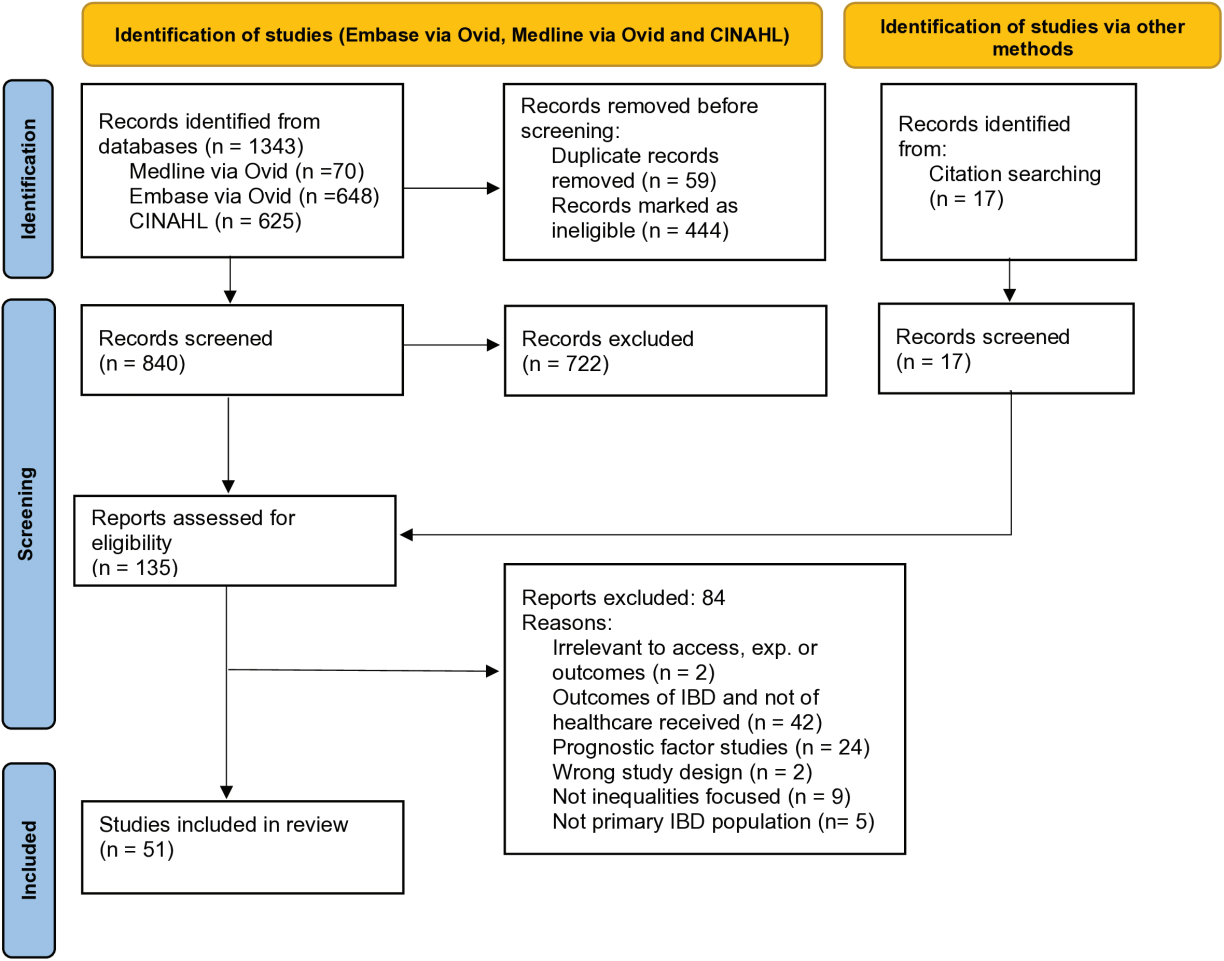
Prisma flow diagram

Two independent reviewers conducted the search and screening process (M.Z. and R.H.). Following the database searches, records were exported to Rayyan.ai (https://www.rayyan.ai/) for screening. In total, 1343 records were screened in Rayyan.ai, and an additional 17 records were identified through additional citation searching (Figure 1 PRISMA flow diagram). Duplicates were detected and removed (*n* = 59), and the PCC criteria were applied to decide if the record was relevant to this review.

### Data Items, Extraction, and Synthesis

Data charting and extraction was conducted by2 researchers (M.Z. and R.H.) and discussed in meetings with a third researcher (D.H.). This phase involved sifting, interpreting, and synthesizing qualitative data from the included articles by tabulating the material.^23^ Discrepancies in agreements were handled through these discussions and clarified by reverting back to the eligibility criteria. A data extraction form was created using an excel spreadsheet to capture the relevant data items for comparison. As recommended by Cochrane guidance,^23^ the form was initially piloted by one researcher (M.Z.) to test its utility. The data items captured were tabulated by the following categories:

- Study information: author(s), publication date, study design, sample size, country, and setting.
- Population: IBD subtype(s), social inequality factors investigated, overall research objective
- Concept: key findings regarding inequalities in accessing care and/or experience of healthcare and/or outcomes of healthcare.

The synthesis of outputs was performed by 3 researchers (M.Z., R.H. and D.H.). A data-driven approach used predetermined themes to guide a narrative synthesis^24^ that described healthcare inequalities relating to (1) patient access, (2) patient experiences, and (3) patient outcomes of IBD. This approach then enabled relationships with and between the studies and the development of a conceptual framework.^24^

We did not conduct a quality appraisal of the included articles, as this is not required of scoping reviews.^18,25^ Study designs were heterogeneous (Table 1). Quality assessment was not included, as this scoping review does not intend to inform clinical decision-making for individuals but to inform health policy makers, service providers, and representatives of service users about areas where underserved IBD groups may need further prioritizing to improve patient access, experience, and outcomes.

## Results

The following results combine the 2 independent searches conducted (M.Z. and R.H.). The final search was conducted on September 27, 2023 (R.H.). A systematic electronic search of the databases (Medline via Ovid, EMBASE via Ovid, and CINAHL) produced a total of 1343 records. Of the 1343 records, 59 duplicate articles were removed, and a further 444 articles were excluded based on their titles that indicated their ineligibility for this review. This left 840 articles for abstract screening; 722 articles were then removed at this stage. Relevant articles, including systematic reviews were also screened in their reference lists to identify additional articles (*n* = 17). One hundred thirty-five articles were subject to full-text screening, and 84 were excluded. This meant that in total, 51 articles were included in this review. Figure 1 presents the PRISMA flowchart and reasons for exclusion of full text articles that were assessed for eligibility.

### Study Characteristics

Fifty-one studies were included with publication dates ranging from 2000 to 2023 (Table 1). Of the included studies, 20 included patients with CD and UC,^26–47^ 12 with UC,^48–59^ 10 with CD,^60–69^ 3 with CD, UC, and Unclassified IBD,^70–72^2 with IBD,^73,74^ and 1 study also referenced “other IBD” amongst UC and CD in their sample.^41^ The majority (*n* = 34) of studies were from the United States,^26,28–36,38,39,43,48–53,55,59–61,63–68,70,72–75^ followed by 7 from the UK,^27,41,42,57,58,69,76^ 5 from Canada,^37,40,45,46,71^ 2 from Sweden,^47,56^ 1 from France,^62^ and 1 from New Zealand.^44^ One study recruited across the United States, Canada, France, and Finland.^54^

**Table 1.** Characteristics of included articles.

Study Author, Year and Country	Study Design	Sample Size	IBD Subtype(S)	Setting	Summary Of Inequality and Demographics (Ethnicity/Race/Age/Gender and Sex/Sexuality/Geographic Location/Other)	Studies Reporting		
						Access	Experience	Outcome
Nguyen et al., (2010), USA	Cross-sectional	286	CD and UC	Hospital	Inequalities in accessing specialist care and medications between “African Americans” and “White Americans”	✔	✔	✔
Mukherjee et al. (2021), UK	Qualitative interviews	33	CD and UC	Community IBD clinic	Experiences of UK South Asian population with IBD of gastroenterology services	✔	✔	✔
Ore et al. (2022), USA	Retrospective cohort	18 742	CD and UC	Clinical database	Postsurgical disparities amongst different ethnic groups including “Black, Asian and Hispanic Whites”			✔
Dos Santos Marques et al. (2020), USA	Retrospective cohort	23 901	CD and UC	Clinical database	Racial disparities in surgical outcomes amongst “Blacks, Asians and Hispanics” compared with “White patients”			✔
Barnes et al. (2021), USA	Retrospective cohort	14 735	CD and UC	Database—4 US states	Racial disparities in medication access between Black and White patients with IBD.	✔		✔
Dos Santos Marques et al. (2022), USA	Qualitative interviews	27	CD and UC	Tertiary IBD center	Understand the surgical experience of Black and White patients with IBD	✔	✔	✔
Montgomery et al. (2018), USA	Retrospective cohort	14 679	CD and UC	Clinical database	Racial disparities in surgical outcomes between Black, White and other patients			✔
Walker et al. (2018), USA	Retrospective cohort	944	CD	Tertiary IBD centre	Income, poverty level and racial inequalities (African Americans and Caucasian Americans) of IBD hospitalizations			✔
Borren et al. (2017), USA	Retrospective cohort	2,136	UC and CD	Tertiary IBD centre	Distance to accessing specialist care and IBD health outcomes	✔		✔
Rubin et al. (2017), USA	Survey	3,608	UC, CD and Unclassified	Community	Inequality in accessing care driven by insurance coverage and affordability of care	✔		✔
Benchimol et al. (2016), Canada	Retrospective cohort	24 192	UC, CD and Unclassified	Clinical database	Inequalities in prediagnosis delay, accessing specialist care and risk of surgery in immigrant populations with IBD	✔		✔
Govani et al. (2016), USA	Retrospective cohort	30 456	UC, CD and Unclassified	Clinical database	Age disparities (elderly > 65) in steroid use and IBD complications			✔
Gunnells et al. (2016), USA	Retrospective cohort	2,523	UC and CD	Clinical database	Disparities in IBD readmissions following colorectal surgery between Black and White patients.			✔
Sewell et al. (2010), USA	Survey	Not reported	UC and CD	National probability sample of ≈ 500 hospitals	Disparities in hospital outcomes between minority patients with IBD	✔		✔
Nguyen et al. (2009), USA	Survey	4,427	CD and UC	Community, hospital and academic medical centers	Racial and geographic inequalities in accessing parenteral nutrition	✔		✔
Li et al. (2008), Sweden	Retrospective cohort	6,552	CD and UC	Clinical database	Influence of education and occupational status on hospitalizations for IBD			✔
Herman et al. (2023), USA	Retrospective cohort	1,462	UC	Clinical database	Ethnic disparities in surgical outcomes following Ileal Pouch Anal Anastomosis			✔
Straus et al. (2000), USA	Cross-sectional survey	145	CD	4 hospitals and 5 private practices	Racial disparities between Black and White patients in access and utilisation of care	✔	✔	✔
Benchimol et al. (2018), Canada	Retrospective cohort	41 879	CD and UC	Clinical database	Disparities in accessing care between rural and urban IBD populations	✔		✔
Nahon et al. (2009), France	Survey	207	CD	Hospital (6)	Disparities in outcomes of care between deprived and non-deprived patients			✔
Frieder et al. (2022), USA	Retrospective cohort	38 143	CD	Clinical database	Racial disparities in patient outcomes that had undergone segmental colectomy			✔
McKenna et al. (2019), USA	Retrospective analysis	4,310	UC	Clinical database	Racial differences in pre and postsurgical outcomes and preventable admissions			✔
Yarur et al. (2014), USA	Case control	142	CD and UC	Hospital	Racial disparities in postoperative complications			✔
Arsoniadis et al. (2017), USA	Retrospective cohort	9513	CD	Clinical database	Racial disparities in postoperative complications			✔
Anyane-Yeboa et al. (2018), USA	Retrospective cohort	203	CD	Hospital	Racial disparities in postoperative recurrence			✔
Cohen-Mekelburg et al. (2019), USA	Retrospective cohort	84	CD	Academic IBD centre	Disparities in delays in commencement of preventable postsurgical biologics between patients with Medicare vs private insurance			✔
Sobotka et al. (2018), USA	Retrospective cohort	4,797	UC	Clinical database	Racial disparities in postoperative outcomes and hospitalization			✔
Olaiya et al. (2020), USA	Retrospective cohort	127	UC	Clinical database	Racial disparities in postoperative outcomes after Colectomy			✔
Jackson et al. (2008), USA	Retrospective cohort	99	CD	Hospital (3)	Racial disparities in health outcomes	✔		✔
Barnes et al. (2018), USA	Prospective cohort	5,537	UC and CD	Academic centers (7)	Racial disparities in the treatment of Black and White patients with IBD			✔
Nguyen et al. (2015), Canada	Retrospective cohort	21 218	UC and CD	Clinical database	Disparities in healthcare utilisation between elderly and young IBD patients	✔		✔
Alexakis et al. (2015), UK	Qualitative interviews	20	CD, UC and other	NHS hospitals (3)	Healthcare challenges faced by Black, Asian and Ethnic Minority patients with IBD	✔	✔	
Li et al. (2014), USA	Retrospective cohort	6,934	UC	Clinical database	Racial disparities in healthcare utilisation within an integrated healthcare organization			✔
Stamatiou et al. (2022), UK	Retrospective cohort	1,620	UC and CD	NHS hospital	Ethnicity/race and socioeconomic disparities in healthcare outcomes			✔
Nguyen et al. (2006), USA	Retrospective cohort	233 389	UC	Clinical database	Racial and geographic disparities in colectomy rates among hospitalised patients.			✔
Nguyen et al. (2007), USA	Retrospective cohort	41 918	CD	Clinical database	Health disparities in CD related bowel resection related to insurance status, ethnicity/race and income			✔
Galooisan et al. (2020), USA	Retrospective cohort	224 500 IBD hospitalizations	UC and CD	Clinical database	Ethnic disparities in IBD-related hospitalization outcome			✔
Kuenzig et al. (2020), Canada	Retrospective cohort	4806	UC and CD	Clinical database	Disparities in accessing care in IBD patients over 65 years old	✔		✔
Odufalu et al. (2023), USA, Canada, France and Finland	Cohort study	1000	UC	Community	Healthcare disparities relating to social determinants, emotional impact and patient experience	✔		
Richard et al. (2020), New Zealand	Qualitative interviews	18	UC and CD	Tertiary IBD centre	Disparities in accessing care for people living in rural New Zealand	✔	✔	
Rohatinsky et al. (2021), Canada	Qualitative interviews	14	UC and CD	Community	Disparities in healthcare utilisation and access to care for rural adults	✔	✔	
Greenstein et al. (2013), USA	Retrospective cohort	2589	UC	Clinical database	Access to care following subtotal Colectomy in UC patients with and without private insurance			✔
Dibley et al. (2014), UK	Qualitative interviews	22	CD and UC	Community	Experiences of healthcare in the gay and lesbian community		✔	
Flasar et al. (2008), USA	Retrospective cohort	406	CD and UC	Medical Centre	Inequalities in access to biologics between African Americans compared with Caucasians.	✔		
Bhurwal et al. (2022), USA	Retrospective cohort	491 451 discharges	UC	Hospital	Inequalities in access to colectomy between different ethnic groups and geographical areas	✔		✔
Nordenvall et al. (2021), Sweden	Retrospective cohort	5969	UC	Clinical database	Socioeconomic drivers of access to surgical care			✔
Farrukh and Mayberry (2015), UK	Retrospective cohort	127	CD	Hospital register	Disparities in access to biologics in CD between European and SA ethnicities	✔		
Farrukh and Mayberry (2016), UK	Retrospective cohort	70	UC	Hospital register	Disparities in access to care in UC between different ethnicities	✔		✔
Farrukh and Mayberry (2022), UK	Retrospective cohort	410	UC	Hospital register	Disparities in access to surgical care in UC between ethnic groups	✔		
Axelrad et al. (2019), USA	Retrospective cohort	947	IBD	Hospital register	Disparities in accessing care in socioeconomically disadvantaged groups			✔
Lin and Sewell (2013), USA	Retrospective cohort	26 400 visits	IBD	Database	Disparities in minority ethnicity/race and socio economic status in accessing emergency care	✔		
					**Total studies**	24	8	42

A range of study designs were included. Thirty-five studies were quantitative retrospective cohorts.^28–30,32–34,37,40,42,43,46–48,50–53,55–61,63–69,71–75^ Five studies utilized survey designs,^35,36,61,62,70^ 6 were qualitative studies using interviews,^27,31,41,44,45,76^ 2 cross-sectional studies,^26,61^ a cohort study,^54^ a case-control design,^38^ and a prospective cohort.^39^ A range of settings for recruiting samples was also used. Nearly half used data available from clinical databases,^28–30,32,34,37,40,43,46–53,56,59,63,64,68,71,72,74^ 4 used hospital registers,^57,58,69,73^ and 1 used national probability samples.^35^ Included studies also recruited through local hospitals,^26,36,38,41,42,55,61,62,65,67^ tertiary IBD centers,^31,33,44,60^ the community,^36,45,54,70,76^ academic IBD centers,^36,39,66^ community IBD clinics,^27^ and private practices.^61^ The lowest sample size across the 51 studies was 14, and the highest was 41 879, with a median of 2136. One study did not report their sample size,^35^ whilst 3 did not report patient sample size but reported emergency department (ED) visits,^74^ hospitalizations,^43^ and hospital discharges.^55^

### Drivers of IBD Inequalities Across the Studies

Table 2 provides a tabulated summary of the inequalities in access, experience, and outcomes reported across the 51 studies. The determinant (eg, race/ethnicity) is given as reported in the research article. Across the included studies, most were focused on race/ethnicity as a driver of inequalities in access, experiences, and/or outcomes of IBD care.^26,28–32,34–36,38,39,42,43,48–53,55,57,58,60,61,63–65,67–70,74,75^ Two studies focused on cultural factors such as immigration^71^ and lack of cultural competence of healthcare systems in driving inequalities in IBD care.^27,41^ Seven studies reported on geographic location or rurality and IBD inequalities,^33,36,37,44,45,53,55^ 6 on socioeconomic markers (income,^54,56,70^ deprivation,^26,42,60,62^ education,^70^ occupation,^47^ and financial stress^70^), to which 6 US studies used health insurance status as a proxy for socioeconomic status.^51,53,55,59,68,70,73^ Other drivers of inequalities investigated included age,^40,70,72^ literacy,^47^ and gender.^54,70^ Only 1 study was found to investigate inequalities in IBD care amongst the LGBTQIA+ population.^76^

### Inequalities in Health Outcomes

Inequality in outcomes for patients with IBD was the most reported in included articles (42 of 51; Table 1). Across many health outcomes, Black ethnic groups fared most poorly. Black or African American ethnic groups were at higher risk of developing postoperative complications,^28,29,32,48,49,63–65^ were more likely to develop IBD-related complications compared with comparative white groups,^39,43^ had greater hospital admissions,^60^ readmissions,^34^ ED attendances,^26,70^ and LOS.^53^ This was found to negatively impact quality of life in this demographic.^61^ Two studies did, however, contradict these findings, showing no evidence of disparities in hospitalizations^26^ and LOS^55^ between Black and white ethnic groups.

Other ethnic groups including “Hispanics” were found to experience postoperative problems^50^ and greater LOS^43^ when compared with white participants^50^ and similar extended LOS was found in an Asian demographic.^29,31^ Other studies found no differences in surgical outcomes between Hispanic and non-Hispanics.^38^ Inflammatory bowel disease patients from “ethnic minority backgrounds” (unspecified) had greater need for intra-abdominal surgery and developed surgical complications within 5 years.^42^ We also found interactions between access, experience, and outcomes. Hispanics experienced greater delays in receiving IBD medical treatments.^36^ Poor outcomes were also found in the South Asian population. Studies found that South Asian individuals with IBD were more likely to conceal their disease, and dietary intake worsened their IBD symptoms.^27^ Further, South Asian patients with UC were over twice as likely to be discharged from hospital compared with patients of European descent.^58^ Studies across different ethnic groups that were not identified as white found lower rates, inferring lower access also of resection^68^ and colectomy.^53^

Socioeconomic factors were also associated with disparate outcomes for patients with IBD. Patients with IBD from more deprived areas and from lower income quartiles had greater ED visits^26^ and hospitalizations.^60,62^ Greater incidence ratios for admissions were also found in people with lower education and for males working in environments exposed to chemicals.^47^ However socioeconomic factors were not found to influence the risks or success of surgery after colectomy.^56^ Some US studies that used medical insurance coverage as a proxy for socioeconomic factors found differing outcomes depending on the type of insurance held by individuals. This meant that some individuals went without treatment to avoid costs.^70^ Delays in treatments were observed amongst those with public insurance (eg, Medicaid) vs private insurance,^66^ as well as increased odds of morbidity and mortality^51,53,68^ and increased hospital admissions.^73^ Further, patients with private insurance were more likely to access colectomy^55,59^ and restorative surgery after colectomy.^56^ No disparities in the use of medications between white and Black ethnic groups were found when patients had similar socioeconomic backgrounds.^30^

Inflammatory bowel disease patients living in more rural areas were found to have increased need for surgery, biological therapies,^33^ and greater hospital admissions^37^ as a result of disease exacerbation. We found fewer reported studies on inequality relating to IBD health outcomes in relation to age, gender, and sexuality. In one study, a greater risk of ED visit was shown for younger (under 40 years) and female patients with IBD.^70^ Age disparities were demonstrated in other studies, which found the elderly IBD population (over 65) were less likely to be prescribed steroids or steroid-sparing drugs^72^ and had less overall healthcare utilization compared with younger IBD patients.^40^ Although another study also found no variation in ED visits, surgical resection or hospitalizations for CD patients over the age of 65.^46^ One study explored health service use and outcomes in an immigrant population with IBD and found no difference in diagnosis, hospitalizations, and ED visits when compared with a nonimmigrant population.^71^ No studies were identified reporting on disparities in outcomes in the LGBTQIA+ population.

### Inequalities in Accessing IBD Healthcare

Approximately half (24 of 51) of the included articles reported inequalities in accessing IBD healthcare (Table 1) and were repeatedly reported in studies of people of Black and South Asian race/ethnicity. Access inequalities in Black and South Asian ethnic groups included lower access to steroids and biological therapies,^26,36,69,75^ reduced access to surgical procedures,^55^ greater difficulties accessing a gastroenterologist,^26,61^ accessing specialist care,^26,58^ and greater use of inpatient services,^35^ although the opposite was found in another study.^67^ Studies also reported inequalities in accessing culturally relevant information for South Asian patients.^27,41^ Other studies also evidenced no disparities in access to medications between Black and white patients with CD^61^ and UC and with similar socioeconomic backgrounds.^30,61,74^ In emergency care settings, no differences were shown in access to biologics when admitted to hospital for Hispanic, Black, and Asian patients with IBD.^74^ Other studies found no disparities in accessing the appropriate surgery when in a flare.^57^ One study found that an immigrant population with IBD had greater access to outpatient and specialist IBD healthcare, suggesting a responsive healthcare system.^71^

Disparities in access were also found in IBD populations living in rural areas. Living further away from tertiary IBD care meant patients had reduced access to specialist IBD care (eg, infusion clinics, hospitals, outpatient clinics) to effectively manage their condition,^33,44,45^ including reduced access to a gastroenterologist^71^ and delayed referrals.^44^

Socioeconomic markers of inequality were also found to impact access to IBD healthcare. The financial impact on individuals meant that patients delayed accessing care due to concerns about cost,^70^ with individuals on lower income also having reduced access to IBD education programs and peer mentoring.^54^ However, one study found no difference between socioeconomic status and access to biologics in emergency care.^74^ Studies also found that the elderly were less likely to access a gastroenterologist or have them as their primary care provider compared with young adults.^40,46^ Males were also less likely to access a gastroenterologist.^54^ There were no studies identified that reported on disparities in access in the LGBTQIA+ population.

### Inequalities in the Experience of IBD Healthcare

Fewer studies (8 of 51) explored inequalities in experiences of IBD care (Table 1). Studies found that Black IBD patients experienced difficulties with scheduling specialist referrals^26^ and delays in appointments due to greater difficulties affording healthcare.^61^ For the South Asian community, patients experienced a lack of cultural understanding and sensitivity of healthcare providers during appointments.^27,41^ This meant parents of young adults with IBD felt limited in their capability to support them.^41^ Rural patients managing their IBD also experienced challenges with healthcare, such as experiencing fragmented care,^45^ describing a feeling of “falling through the cracks.”^44^ One study explored experiences of LGBTQIA+ individuals with IBD, which found that individuals felt as though they were treated differently compared with other patients and lacked acknowledgment of their partners during consultations.^76^

**Figure 2.**
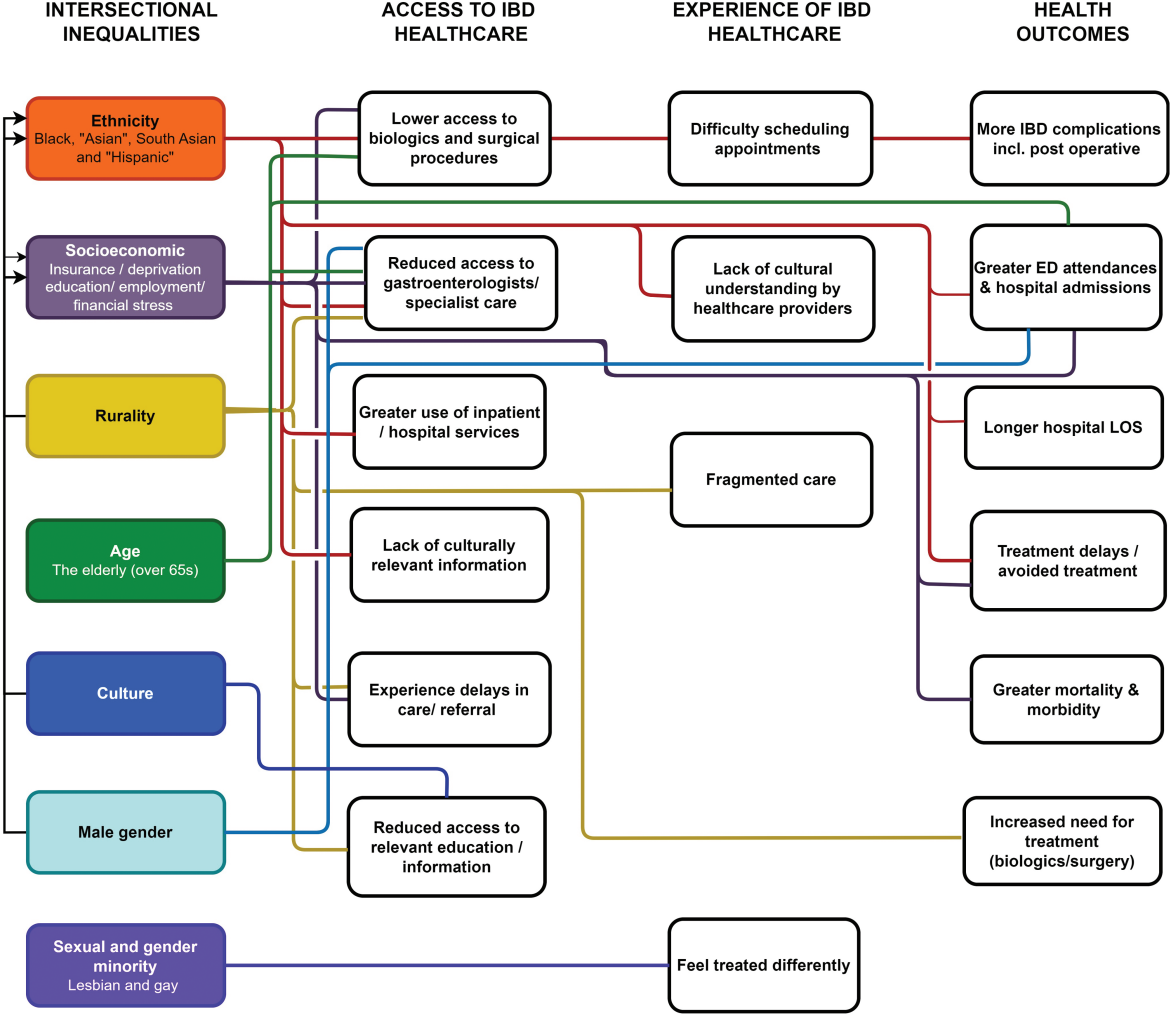
Inflammatory bowel disease healthcare inequalities in access, experience, and outcomes. A description of the inequalities identified across the included articles of this review. Color coding signifies each inequality variable. Lines indicate a positive relationship between the constructs. Arrowed lines indicate intersectionality between inequality variables.

Figure 2 illustrates the relationships between the intersectional factors of inequalities and access, experience, and outcomes.

## Discussion

Fifty-one studies were incorporated into this review in which the majority (42 of 51) were focused on inequalities in IBD health outcomes, followed by access (24 of 51) and experiences of healthcare (8 of 51). We identified articles that explored healthcare inequalities between different ethnic/racial groups, cultural factors, distance to healthcare, and rurality and socioeconomic factors. Under-researched areas of inequalities in IBD healthcare-covered factors include age, literacy, gender and sex, and sexual and gender minority individuals. We found no studies understanding perspectives from bisexual, transgender, queer, pansexual, or asexual+ individuals. This is the first scoping review that synthesizes inequities in IBD care driven across such a wide range of social inequality factors. This review expands upon previous review studies that have confirmed inequalities in access and outcomes of IBD healthcare.^13,17,77^ It provides a more up to date perspective of such inequalities in access and outcomes and also incorporates more studies focusing on inequalities of experience. However, we highlight that a significant gap remains in research understanding disparities in experience of IBD healthcare compared with access and outcomes. Fewer studies were identified examining the impact of inequality on patient experience. This may relate to a limited recognition of experience as a concept and limited tools that measure experience of care in IBD.^78^

A greater body of research was found to evidence inequalities in access, experience, and outcomes of IBD care across Black, Asian, and Hispanic (in US studies) ethnic groups compared with white or counterparts of European descent. We found studies showing poorer health outcomes including greater rate of IBD complications,^28,29,32,39,42,43,48,49,63–65^ ED attendances, and hospital admissions.^26,34,60,70^ Studies also found reduced access to biological and surgical treatments^26,36,55,69,75^ and specialist gastroenterology care^26,58,61^ in Black and South Asian individuals with IBD. These findings are unsurprising given evidence across healthcare showing ethnic inequalities in accessing appropriate information, digital exclusion,^79^ and experiences of discrimination and alienation across mental^80^ and physical health services.^81^ Other reviews focusing on ethnic/racial disparities in IBD healthcare utilization have also noted such disparities.^13^ This supports the need for cultural humility in healthcare to appropriately support access, experience, and health outcomes for underserved ethnic groups living with IBD.^27,41^ Cultural humility is defined as providing care to patients with openness to power-sharing, that is self-reflective, and has an understanding of the patients experience—or lack of—with healthcare.^82^ It also fits with access being a complex construct that reflects “best fit” between service and service user.^83^ A shift to cultural humility and safety in healthcare over cultural competence is reflective of the argued limited impact on reducing health inequalities of cultural competence. Cultural competence frames the understanding of culture towards individuals rather than reflective assessment of power and bias.^84–86^

Poorer healthcare access and subsequent worsened health outcomes were also observed for IBD patients from deprived backgrounds. Individuals on lower income or experiencing financial distress were found to experience reduced access to IBD information^54^ and delayed access to care,^70^ including surgical procedures^55,56,59^ and therefore had greater ED visits^26^ and hospitalizations.^60,62^ Research also finds that the most economically vulnerable populations have greater rates of avoidable emergency admissions outside of the IBD population.^87^ However, no studies explored patient experiences of IBD care in deprived communities—again warranting further research in this area. Distance to healthcare and rurality was explored across 7 studies of this review, which found similar disparities in access and outcomes. More nuanced experiences of healthcare for rural individuals using qualitative methodology described experiences of fragmented care.^44,45^ Only 6 qualitative studies were identified in this review, leaving a discernible gap in understanding experiences of IBD healthcare inequalities. We recommend further qualitative research in this area.

Whilst findings from this review recognize that race/ethnicity, rurality, and socioeconomic factors are driving IBD healthcare inequalities, research is lacking in understanding other groups vulnerable to healthcare inequalities. Two studies in this review evidenced inequalities for males with IBD. Males working in chemically exposing environments had greater incidences of admissions^47^ and were also less likely to have access to a gastroenterologist.^54^ Whilst few studies in this review highlight this inequality, other research also shows that men are lower utilizers of healthcare and are more likely to adopt riskier health behaviors^88,89^ that increase their risk of noncommunicable diseases including cardiovascular disease and cancer.^90^ However, whilst older studies have found reduced medication adherence in males with IBD,^91^ more recent studies have found this is greater in younger women.^92^

Only one study was found to explore experiences of healthcare in the LGBTQIA+ population^76^. A lack of IBD specific research understanding experiences in the LGBTQIA+ community is echoed in other studies.^93–95^ Epidemiological studies from the United States demonstrate the higher prevalence rates of CD and UC in men who have sex with men compared with heterosexual males with similar high-risk sexual activity.^95^ We know that inequalities in care access, health outcomes, and in experiencing discrimination and stigma are common amongst patient groups identifying as LGBTQIA+ across clinical care ^96^. As such, there is therefore a clear need for more research with this community to inform service configuration and delivery for LGBTQIA+ individuals. We found no studies addressing healthcare inequalities in individuals with learning disabilities and autism—surprising given the prevalence of learning disabilities and autism in the IBD population.^97^ This was also true for studies addressing other physical disabilities comorbid to IBD with no articles identified in the search. This included additional Google scholar and PubMed searches, undertaken for completeness. There is therefore a significant gap in research to understand health inequalities in individuals with IBD and comorbid learning and physical disabilities. Focusing on particular population groups over others risks creating inequalities within inequalities.^14^ Therefore, research in each of these marginalized communities with IBD is important.

An important finding is the significant interaction between factors influencing inequality—and which frequently coexist in the community. For example, studies found that Black ethnic individuals worried over the costs of healthcare^26,61^ and difficulties travelling to appointments,^61^ which together impact accessibility and health outcomes. More economically deprived Black patients experienced greater numbers of ED visits.^26,60^ Similarly, health outcomes such as risk of postoperative complications differed between the most and least deprived Black, Hispanic, and Asian/Pacific Islanders individuals with IBD.^68^ An intersectional view of inequalities might help avoid a reductive view by taking into account the interacting social and system factors that impact overall health and well-being.^98^ American studies have shown that older Black ethnic populations with IBD have disproportionately lower health literacy.^99^ Other possible interacting factors impacted access, experience, and outcomes of IBD care may also include language barriers. Challenges in accessing translation services for South Asian patients with IBD were found in a qualitative study understanding experiences of UK gastroenterology services.^27^ Similar findings are demonstrated in other UK studies with Bangladeshi native-speaking patients with IBD, further widening inequalities in health.^100^ Therefore, further research adopting an intersectional lens of health is needed to uncover interacting inequalities in IBD access, experience, and outcomes of healthcare.

The findings of this review are important for clinicians and in the organization of services. Recommendations in South Asian patients have included improved psychological support and translation support, targeted dietary advice, and measures to increase awareness in that population.^101^ However, this review clearly demonstrates the need for measures across all groups where inequality is an issue. This may need action based on the findings but also local initiatives with individual communities to understand issues of importance to those communities.

### Strengths and Limitations

To the authors’ knowledge, this is the only review synthesizing the IBD literature of inequalities across healthcare access, experience, and outcomes. Previous studies have focused on inequalities in health outcomes,^17^ but not experiences and access. The findings presented in this review are aggregated from across 7 countries, with the majority originating from the United States (34 of 51). From a UK perspective, only 7 studies met the eligibility criteria, indicating the need for future UK-focused research exploring healthcare inequalities in IBD. Systemic healthcare differences in the US vs the UK National Health Service (NHS) should be considered when interpreting results, such as the insurance system mechanisms which exacerbate health inequalities. However, similarities to the Core20PLUS5 initiative from NHS England^16^ show that vulnerable groups (eg, the most deprived populations and particular ethnic groups) experience inequalities in healthcare access, experience, and outcomes and therefore are comparable for literature reviews.

We acknowledge our use of “race/ethnicity” in the reporting of this review. Race and ethnicity are defined as social constructs—with the terms and categories describing them having changed over time as a result of racism and sociocultural shifts in society.^102^ There are differences in the use of these terms across different countries investigating health inequalities. Nevertheless, use of categories for ethnicity and race may be very real in their impact in reinforcing inequality. Caution is needed when interpreting the race/ethnicity and heritage of populations included in this review. Many studies did not report the specific diversity within populations. These have been included as in similar systematic reviews.^11^

We also found that many articles in this review did not explicitly define or differentiate between gender and sex. Guidelines emphasize the need to define whether sex and gender is captured and whether this was self-reported or assigned via medical records.^103^

There is also an interaction between the impacts of inequality that have been assessed in this review that may not be separated in publications. For example, it is likely that impaired access will result in poorer outcomes and experience of care.

The limitations of conducting a scoping review without a quality appraisal of the included articles is also acknowledged. For example, in one study, we found no evidence of disparities in IBD biologics including immunomodulators, antitumor necrosis factor therapies and combination therapies between white and Black ethnic groups in patients with similar socioeconomic backgrounds.^30^ However, we acknowledge that it may have been the study design or quality may have contributed to this finding.

## Conclusion

This review collated published evidence on healthcare inequalities in access, experience, and outcomes of IBD care. Research and service changes should address these inequalities. There has been greater attention to understanding unequal health outcomes, with less focus on experiences and access to care. Very few qualitative studies exist exploring healthcare experiences across underserved populations with IBD. Inequalities relating to race/ethnicity are most reported, demonstrating significant inequities for Black, Asian, and Hispanic ethnic groups and emphasizing the need for cultural humility in IBD healthcare. Inflammatory bowel disease patients living in deprivation, on lower income, and living further away from specialist IBD care also experience healthcare inequalities. However, significant gaps exist in understanding healthcare experiences and outcomes for IBD patients who identify as LGBTQIA+ and also with learning disabilities or autism. The lack of research in this area poses a risk of creating inequalities within inequalities.

## Supplementary Material

izae077_suppl_Supplementary_Material_S1

izae077_suppl_Supplementary_Material_S2

izae077_suppl_Supplementary_Material_S3

izae077_suppl_Supplementary_Material_S4

## References

[1] Seyedian SS , Nokhostin F, Malamir MD. A review of the diagnosis, prevention, and treatment methods of inflammatory bowel disease. J Med Life. 2019;12(2):113-122. 31406511 10.25122/jml-2018-0075 PMC6685307

[2] Lamb CA , Kennedy NA, Raine T, et al; IBD guidelines eDelphi consensus group. British Society of Gastroenterology consensus guidelines on the management of inflammatory bowel disease in adults. Gut. 2019;68(Suppl 3):s1-s106. 31562236 10.1136/gutjnl-2019-318484 PMC6872448

[3] Fudman DI , Perez-Reyes AE, Niccum BA, Melmed GY, Khalili H. Interventions to decrease unplanned healthcare utilization and improve quality of care in adults with inflammatory bowel disease: a systematic review. Clin Gastroenterol Hepatol. 2022;20(9):1947-1970.e7. 34481951 10.1016/j.cgh.2021.08.048

[4] Deepak P , Barnes EL, Shaukat A. Health disparities in inflammatory bowel disease care driven by rural versus urban residence: challenges and potential solutions. Clin Gastroenterol Hepatol. 2023;21(7):1681-1686. 37353301 10.1016/j.cgh.2023.04.006

[5] Pathiyil MM , Jena A, Venkataramana Raju AK, et al Representation and reporting of diverse groups in randomised controlled trials of pharmacological agents in inflammatory bowel disease: a systematic review. Lancet Gastroenterol Hepatol. 2023;8(12):1143-1151. doi: https://doi.org/10.1016/S2468-1253(23)00193-0 37832569

[6] Marmot M. Fair Society, Healthy Lives: The Marmot Review. Strategic Review of Health Inequalities in England Post-2010. The Marmot Review; 2010.

[7] NHS England. What are healthcare inequalities? NHS England. Accessed November 1, 2023. https://www.england.nhs.uk/about/equality/equality-hub/national-healthcare-inequalities-improvement-programme/what-are-healthcare-inequalities/

[8] World Health Organisation. Closing the gap in a generation: health equity through action on the social determinants of health: commission on social determinants of health final report. World Health Organisation, 2008. Accessed November 1, 2023. https://play.google.com/store/books/details?id=zc_VfH7wfV8C

[9] Arcaya MC , Arcaya AL, Subramanian SV. Inequalities in health: definitions, concepts, and theories. Glob Health Action. 2015;8(1):27106. 26112142 10.3402/gha.v8.27106 PMC4481045

[10] Farrukh A , Mayberry J. Apparent disparities in hospital admission and biologic use in the management of inflammatory bowel disease between 2014–2018 in some black and ethnic minority (BEM) populations in England. Gastrointest Disord. 2020;2(2):144-151.

[11] Ahmed S , Newton PD, Ojo O, Dibley L. Experiences of ethnic minority patients who are living with a primary chronic bowel condition: a systematic scoping review with narrative synthesis. BMC Gastroenterol. 2021;21(1):322. 34407752 10.1186/s12876-021-01857-8 PMC8371833

[12] Wardle RA , Wardle AJ, Charadva C, Ghosh S, Moran GW. Literature review: impacts of socioeconomic status on the risk of inflammatory bowel disease and its outcomes. Eur J Gastroenterol Hepatol. 2017;29(8):879-884. 28471825 10.1097/MEG.0000000000000899

[13] Tandon P , Chhibba T, Natt N, et al Significant racial and ethnic disparities exist in health care utilization in inflammatory bowel disease: a systematic review and meta-analysis. Inflamm Bowel Dis. 2023;30(3):470-481. 10.1093/ibd/izad045 36975373

[14] Ford J , Sowden S, Olivera J, et al Transforming health systems to reduce health inequalities. Future Healthc J. 2021;8(2):e204-e209. 34286186 10.7861/fhj.2021-0018 PMC8285147

[15] Farrukh A , Mayberry JF. NHS trust boards and health and well-being boards: do they play any role in the management of disparate levels of care for South Asian patients with inflammatory bowel disease? Ulster Med J. 2023;92(1):38-42. 36762141 PMC9899024

[16] NHS. Core20PLUS5 (adults) – an approach to reducing healthcare inequalities. NHS England. 2022. Accessed January 4, 2023. https://www.england.nhs.uk/about/equality/equality-hub/national-healthcare-inequalities-improvement-programme/core20plus5/

[17] Booth A , Ford W, Brennan E, et al Towards equitable surgical management of inflammatory bowel disease: a systematic review of disparities in surgery for inflammatory bowel disease. Inflamm Bowel Dis. 2022;28(9):1405-1419. 34553754 10.1093/ibd/izab237

[18] Tricco AC , Lillie E, Zarin W, et al PRISMA extension for scoping reviews (PRISMA-ScR): checklist and explanation. Ann Intern Med. 2018;169(7):467-473. 30178033 10.7326/M18-0850

[19] Arksey H , O’Malley L. Scoping studies: towards a methodological framework. Int J Soc Res Methodol. 2005;8(1):19-32.

[20] Peters M , Godfrey C, McInerney P, Soares C, Khalil H, Parker D. Methodology for JBI Scoping Reviews. The Joanna Briggs Institute; 2015.

[21] Borrell LN , Elhawary JR, Fuentes-Afflick E, et al Race and genetic ancestry in medicine - a time for reckoning with racism. N Engl J Med. 2021;384(5):474-480. 33406325 10.1056/NEJMms2029562 PMC8979367

[22] Napier AD , Ancarno C, Butler B, et al Culture and health. Lancet. 2014;384(9954):1607-1639. 25443490 10.1016/S0140-6736(14)61603-2

[23] Higgins JPT. *Cochrane handbook for systematic reviews of interventions version 5.0. 1. The Cochrane Collaboration* . 2008. Accessed January 5, 2024. http://www cochrane-handbookorg.

[24] Popay J , Roberts HM, Sowden AJ, et al Guidance on the Conduct of Narrative Synthesis in Systematic Reviews. A Product from the ESRC Methods Programme. Version 1. 2006.

[25] Peters MDJ , Godfrey CM, Khalil H, et al Guidance for conducting systematic scoping reviews. Int J Evid Based Healthc. 2015;13(3):141-146. 26134548 10.1097/XEB.0000000000000050

[26] Nguyen GC , LaVeist TA, Harris ML, et al Racial disparities in utilization of specialist care and medications in inflammatory bowel disease. Am J Gastroenterol. 2010;105(10):2202-2208. 20485281 10.1038/ajg.2010.202 PMC3170037

[27] Mukherjee S , Beresford B, Atkin K, Sebastian S. The need for culturally competent care within gastroenterology services: evidence from research with adults of south asian origin living with inflammatory bowel disease. J Crohns Colitis. 2021;15(1):14-23. 32577761 10.1093/ecco-jcc/jjaa117

[28] Ore AS , Vigna C, Fabrizio A, Messaris E. Evaluation of racial/ethnic disparities in the surgical management of inflammatory bowel disease. J Gastrointest Surg. 2022;26(12):2559-2568. 36253503 10.1007/s11605-022-05483-x

[29] Dos Santos Marques IC , Theiss LM, Wood LN, et al Racial disparities exist in surgical outcomes for patients with inflammatory bowel disease. Am J Surg. 2020;221(4):668-674. 33309255 10.1016/j.amjsurg.2020.12.010

[30] Barnes EL , Bauer CM, Sandler RS, Kappelman MD, Long MD. Black and white patients with inflammatory bowel disease show similar biologic use patterns with Medicaid insurance. Inflamm Bowel Dis. 2021;27(3):364-370. 32405642 10.1093/ibd/izaa090 PMC7885313

[31] Dos Santos Marques IC , Herbey II, Theiss LM, et al Understanding the surgical experience for Black and White patients with inflammatory bowel disease (IBD): The importance of health literacy. Am J Surg. 2021;223(2):303-311. 34119329 10.1016/j.amjsurg.2021.06.003 PMC8655316

[32] Montgomery SR, Jr, Butler PD, Wirtalla CJ, et al Racial disparities in surgical outcomes of patients with inflammatory bowel disease. Am J Surg. 2018;215(6):1046-1050. 29803499 10.1016/j.amjsurg.2018.05.011 PMC7764563

[33] Borren NZ , Conway G, Tan W, et al Distance to specialist care and disease outcomes in inflammatory bowel disease. Inflamm Bowel Dis. 2017;23(7):1234-1239. 28520589 10.1097/MIB.0000000000001133 PMC5531761

[34] Gunnells DJ , Morris MS, DeRussy A, et al Racial disparities in readmissions for patients with inflammatory bowel disease (IBD) after colorectal surgery. J Gastrointest Surg. 2016;20(5):985-993. 26743885 10.1007/s11605-015-3068-9

[35] Sewell JL , Yee HF, Jr, Inadomi JM. Hospitalizations are increasing among minority patients with Crohn’s disease and ulcerative colitis. Inflamm Bowel Dis. 2010;16(2):204-207. 19575353 10.1002/ibd.21008

[36] Nguyen GC , Munsell M, Brant SR, LaVeist TA. Racial and geographic disparities in the use of parenteral nutrition among inflammatory bowel disease inpatients diagnosed with malnutrition in the United States. JPEN J Parenter Enteral Nutr. 2009;33(5):563-568. 19564625 10.1177/0148607109332907 PMC2962862

[37] Benchimol EI , Kuenzig ME, Bernstein CN, et al; Canadian Gastro-Intestinal Epidemiology Consortium. Rural and urban disparities in the care of Canadian patients with inflammatory bowel disease: a population-based study. Clin Epidemiol. 2018;10:1613-1626. 30519110 10.2147/CLEP.S178056 PMC6233859

[38] Yarur AJ , Abreu MT, Salem MS, Deshpande AR, Sussman DA. The impact of Hispanic ethnicity and race on post-surgical complications in patients with inflammatory bowel disease. Dig Dis Sci. 2014;59(1):126-134. 23483313 10.1007/s10620-013-2603-3

[39] Barnes EL , Kochar B, Long MD, et al Lack of difference in treatment patterns and clinical outcomes between black and white patients with inflammatory bowel disease. Inflamm Bowel Dis. 2018;24(12):2634-2640. 29788063 10.1093/ibd/izy179 PMC6262194

[40] Nguyen GC , Sheng L, Benchimol EI. Health care utilization in elderly onset inflammatory bowel disease: a population-based study. Inflamm Bowel Dis. 2015;21(4):777-782. 25738376 10.1097/MIB.0000000000000306

[41] Alexakis C , Nash A, Lloyd M, et al Inflammatory bowel disease in young patients: challenges faced by black and minority ethnic communities in the UK. Health Soc Care Community. 2015;23(6):665-672. 25660726 10.1111/hsc.12188

[42] Stamatiou D , Naumann DN, Foss H, Singhal R, Karandikar S. Effects of ethnicity and socioeconomic status on surgical outcomes from inflammatory bowel disease. Int J Colorectal Dis. 2022;37(6):1367-1374. 35554640 10.1007/s00384-022-04180-0

[43] Galoosian A , Rezapour M, Liu B, Bhuket T, Wong RJ. Race/ethnicity-specific disparities in in-hospital mortality and hospital charges among inflammatory bowel disease-related hospitalizations in the United States. J Clin Gastroenterol. 2020;54(7):e63-e72. 31008866 10.1097/MCG.0000000000001204

[44] Richard L , Geoff N, Sarah D, et al Patients’ accounts of living with and managing inflammatory bowel disease in rural Southern New Zealand: a qualitative study. BMJ Open. 2020;10(11):e041789. 10.1136/bmjopen-2020-041789 PMC7662529 33184085

[45] Rohatinsky N , Boyd I, Dickson A, et al Perspectives of health care use and access to care for individuals living with inflammatory bowel disease in rural Canada. Rural Remote Health. 2021;21(2):6358. 33820422 10.22605/RRH6358

[46] Kuenzig ME , Stukel TA, Kaplan GG, et al Variation in care of patients with elderly-onset inflammatory bowel disease in Ontario, Canada: a population-based cohort study. J Can Assoc Gastroenterol. 2021;4(2):e16-e30. 33855268 10.1093/jcag/gwz048 PMC8023856

[47] Li X , Sundquist J, Sundquist K. Educational level and occupation as risk factors for inflammatory bowel diseases: a nationwide study based on hospitalizations in Sweden. Inflamm Bowel Dis. 2009;15(4):608-615. 19067408 10.1002/ibd.20815

[48] Herman K , Pokala A, Nemeth S, Shen B. Ethnic disparities in ileal pouch anal anastomosis outcomes: an ACS-NSQIP Study. J Surg Res. 2022;283:84-92. 36395743 10.1016/j.jss.2022.09.024

[49] McKenna NP , Habermann EB, Glasgow AE, Mathis KL, Lightner AL. Risk factors for readmission following ileal pouch-Anal anastomosis: an American College of Surgeons National Surgical Quality Improvement Program analysis. J Surg Res. 2018;229:324-331. 29937009 10.1016/j.jss.2018.04.037

[50] Sobotka LA , Husain SG, Krishna SG, et al A risk score model of 30-day readmission in ulcerative colitis after colectomy or proctectomy. Clin Transl Gastroenterol. 2018;9(8):175. 30108206 10.1038/s41424-018-0039-y PMC6092348

[51] Olaiya B , Renelus BD, Filon M, Saha S. Trends in morbidity and mortality following colectomy among patients with ulcerative colitis in the biologic era (2002-2013): a study using the national inpatient sample. Dig Dis Sci. 2021;66(6):2032-2041. 32676826 10.1007/s10620-020-06474-1

[52] Li D , Collins B, Velayos FS, et al Racial and ethnic differences in health care utilization and outcomes among ulcerative colitis patients in an integrated health-care organization. Dig Dis Sci. 2014;59(2):287-294. 24173809 10.1007/s10620-013-2908-2

[53] Nguyen GC , Laveist TA, Gearhart S, Bayless TM, Brant SR. Racial and geographic variations in colectomy rates among hospitalized ulcerative colitis patients. Clin Gastroenterol Hepatol. 2006;4(12):1507-1513. 17162242 10.1016/j.cgh.2006.09.026

[54] Odufalu FD , Dubinsky MC, Peyrin-Biroulet L, et al Health care disparities, social determinants of health, and emotional impacts in patients with ulcerative colitis: results from a global ulcerative colitis narrative patient survey. Inflamm Bowel Dis. 2023;29(11):1681-1692. 37300505 10.1093/ibd/izad102 PMC10628921

[55] Bhurwal A , Minacapelli CD, Patel A, et al Evaluation of a U.S. national cohort to determine utilization in colectomy rates for ulcerative colitis among ethnicities. Inflamm Bowel Dis. 2022;28(1):54-61. 33534892 10.1093/ibd/izab020

[56] Nordenvall C , Westberg K, Söderling J, et al Restorative surgery is more common in ulcerative colitis patients with a high income: a population-based study. Dis Colon Rectum. 2021;64(3):301-312. 33395139 10.1097/DCR.0000000000001775

[57] Farrukh A , Mayberry JF. Surgery for ulcerative colitis in the White British and South Asian populations in selected trusts in England 2001-2020: an absence of disparate care and a need for specialist centres. J Clin Med Res. 2022;11(17):4967. doi: https://doi.org/10.3390/jcm11174967 PMC9457178 36078897

[58] Farrukh A , Mayberry J. Patients with ulcerative colitis from diverse populations: the leicester experience. Med Leg J. 2016;84(1):31-35. 26078265 10.1177/0025817215590769

[59] Greenstein AJ , Romanoff AM, Moskowitz AJ, et al Payer status and access to laparoscopic subtotal colectomy for ulcerative colitis. Dis Colon Rectum. 2013;56(9):1062-1067. https://journals.lww.com/dcrjournal/fulltext/2013/09000/payer_status_and_access_to_laparoscopic_subtotal.7.aspx 23929015 10.1097/DCR.0b013e31829b2d30

[60] Walker C , Allamneni C, Orr J, et al Socioeconomic status and race are both independently associated with increased hospitalization rate among Crohn’s disease patients. Sci Rep. 2018;8(1):4028. 29507339 10.1038/s41598-018-22429-z PMC5838155

[61] Straus WL , Eisen GM, Sandler RS, Murray SC, Sessions JT. Crohn’s disease: does race matter? The Mid-Atlantic Crohn’s Disease Study Group. Am J Gastroenterol. 2000;95(2):479-483. 10685754 10.1111/j.1572-0241.2000.t01-1-01531.x

[62] Nahon S , Lahmek P, Macaigne G, et al Socioeconomic deprivation does not influence the severity of Crohn’s disease: results of a prospective multicenter study. Inflamm Bowel Dis. 2009;15(4):594-598. 19085998 10.1002/ibd.20794

[63] Frieder JS , Montorfano L, De Stefano F, et al A national inpatient sample analysis of racial disparities after segmental colectomy for inflammatory colorectal diseases. Am Surg. 2022;89(12):5131-5139. 36349487 10.1177/00031348221138085

[64] Arsoniadis EG , Ho YY, Melton GB, Madoff RD, Le C, Kwaan MR. African Americans and short-term outcomes after surgery for Crohn’s Disease: an ACS-NSQIP analysis. J Crohns Colitis. 2017;11(4):468-473. 27683803 10.1093/ecco-jcc/jjw175 PMC5881719

[65] Anyane-Yeboa A , Yamada A, Haider H, et al A comparison of the risk of postoperative recurrence between African-American and Caucasian patients with Crohn’s disease. Aliment Pharmacol Ther. 2018;48(9):933-940. 30126019 10.1111/apt.14951 PMC6669906

[66] Cohen-Mekelburg S , Gold S, Schneider Y, et al Delays in initiating post-operative prophylactic biologic therapy are common among Crohn’s disease patients. Dig Dis Sci. 2019;64(1):196-203. 29876778 10.1007/s10620-018-5159-4

[67] Jackson JF, 3rd, Dhere T, Repaka A, Shaukat A, Sitaraman S. Crohn’s disease in an African-American population. Am J Med Sci. 2008;336(5):389-392. 19011394 10.1097/MAJ.0b013e31816a5c06

[68] Nguyen GC , Bayless TM, Powe NR, LaVeist TA, Brant SR. Race and health insurance are predictors of hospitalized Crohn’s disease patients undergoing bowel resection. Inflamm Bowel Dis. 2007;13(11):1408-1416. 17567876 10.1002/ibd.20200

[69] Farrukh A , Mayberry JF. Apparent discrimination in the provision of biologic therapy to patients with Crohn’s disease according to ethnicity. Public Health. 2015;129(5):460-464. 25779216 10.1016/j.puhe.2015.01.029

[70] Rubin DT , Feld LD, Goeppinger SR, et al The Crohn’s and colitis foundation of America survey of inflammatory bowel disease patient health care access. Inflamm Bowel Dis. 2017;23(2):224-232. 27997434 10.1097/MIB.0000000000000994

[71] Benchimol EI , Manuel DG, Mojaverian N, et al Health services utilization, specialist care, and time to diagnosis with inflammatory bowel disease in immigrants to Ontario, Canada: a population-based cohort study. Inflamm Bowel Dis. 2016;22(10):2482-2490. 27556836 10.1097/MIB.0000000000000905

[72] Govani SM , Wiitala WL, Stidham RW, et al Age disparities in the use of steroid-sparing therapy for inflammatory bowel disease. Inflamm Bowel Dis. 2016;22(8):1923-1928. 27416039 10.1097/MIB.0000000000000817 PMC4956567

[73] Axelrad JE , Sharma R, Laszkowska M, et al Increased healthcare utilization by patients with inflammatory bowel disease covered by Medicaid at a tertiary care center. Inflamm Bowel Dis. 2019;25(10):1711-1717. 30989212 10.1093/ibd/izz060 PMC7327156

[74] Lin KK , Sewell JL. The effects of race and socioeconomic status on immunomodulator and anti-tumor necrosis factor use among ambulatory patients with inflammatory bowel disease in the United States. Am J Gastroenterol. 2013;108(12):1824-1830. 24300857 10.1038/ajg.2013.192

[75] Flasar MH , Johnson T, Roghmann MC, Cross RK. Disparities in the use of immunomodulators and biologics for the treatment of inflammatory bowel disease: a retrospective cohort study. Inflamm Bowel Dis. 2008;14(1):13-19. 17973305 10.1002/ibd.20298

[76] Dibley L , Norton C, Schaub J, Bassett P. Experiences of gay and lesbian patients with inflammatory bowel disease: a mixed methods study. Gastrointest Nurs. 2014;12(6):19-30.

[77] Sewell JL , Velayos FS. Systematic review: the role of race and socioeconomic factors on IBD healthcare delivery and effectiveness. Inflamm Bowel Dis. 2013;19(3):627-643. 22623078 10.1002/ibd.22986 PMC3905682

[78] Sheldon EM , Lillington G, Simpson K, et al Development of an inflammatory bowel disease (IBD) Patient-Reported Experience Measure (PREM): a patient-led consensus work and “think aloud” study for a quality improvement programme. Health Expect. 2022;26(1):213-225. 36335578 10.1111/hex.13647 PMC9854292

[79] Woolley KE , Bright D, Ayres T, et al Mapping inequities in digital health technology within the World Health Organization’s European region using PROGRESS PLUS: scoping review. J Med Internet Res. 2023;25:e44181. 37115613 10.2196/44181 PMC10182469

[80] Lawrence V , McCombie C, Nikolakopoulos G, Morgan C. Ethnicity and power in the mental health system: experiences of white British and black Caribbean people with psychosis. Epidemiol Psychiatr Sci. 2021;30(5):e12. 33543688 10.1017/S2045796020001043 PMC8057456

[81] Kapadia D , Jingwen Z, Salway S, et al Ethnic Inequalities in Healthcare: A Rapid Evidence Review. NHS Race & Health Observatory; 2022.

[82] Lekas HM , Pahl K, Fuller Lewis C. Rethinking cultural competence: shifting to cultural humility. Health Serv Insights. 2020;13:1178632920970580. 33424230 10.1177/1178632920970580 PMC7756036

[83] Voorhees J , Bailey S, Waterman H, Checkland K. Accessing primary care and the importance of “human fit”: a qualitative participatory case study. Br J Gen Pract. 2022;72(718):e342-e350. 34990392 10.3399/BJGP.2021.0375 PMC8843400

[84] Curtis E , Jones R, Tipene-Leach D, et al Why cultural safety rather than cultural competency is required to achieve health equity: a literature review and recommended definition. Int J Equity Health. 2019;18(1):174. 31727076 10.1186/s12939-019-1082-3 PMC6857221

[85] Shen Z. Cultural competence models and cultural competence assessment instruments in nursing: a literature review. J Transcult Nurs. 2015;26(3):308-321. 24817206 10.1177/1043659614524790

[86] Kaihlanen AM , Hietapakka L, Heponiemi T. Increasing cultural awareness: qualitative study of nurses’ perceptions about cultural competence training. BMC Nurs. 2019;18(1):38. 31440116 10.1186/s12912-019-0363-x PMC6704569

[87] King JA , Underwood FE, Panaccione N, et al Trends in hospitalisation rates for inflammatory bowel disease in western versus newly industrialised countries: a population-based study of countries in the Organisation for Economic Co-operation and Development. Lancet Gastroenterol Hepatol. 2019;4(4):287-295. 30765267 10.1016/S2468-1253(19)30013-5

[88] Ashton LM , Hutchesson MJ, Rollo ME, Morgan PJ, Collins CE. A scoping review of risk behaviour interventions in young men. BMC Public Health. 2014;14(1):957. 25224717 10.1186/1471-2458-14-957 PMC4177699

[89] Pinkhasov RM , Wong J, Kashanian J, et al Are men shortchanged on health? Perspective on health care utilization and health risk behavior in men and women in the United States. Int J Clin Pract. 2010;64(4):475-487. 20456194 10.1111/j.1742-1241.2009.02290.x

[90] Zwolinsky S , Raine G, Robertson S. Prevalence, Co-occurrence and clustering of lifestyle risk factors among UK men. J Men’s Health. 2016;12(2):15-24. doi: https://doi.org/10.31083/jomh.v12i2.24

[91] Hawthorne AB , Rubin G, Ghosh S. Review article: medication non-adherence in ulcerative colitis--strategies to improve adherence with mesalazine and other maintenance therapies. Aliment Pharmacol Ther. 2008;27(12):1157-1166. 18384664 10.1111/j.1365-2036.2008.03698.x

[92] Lopez A , Billioud V, Peyrin-Biroulet C, Peyrin-Biroulet L. Adherence to anti-TNF therapy in inflammatory bowel diseases: a systematic review. Inflamm Bowel Dis. 2013;19(7):1528-1533. 23518810 10.1097/MIB.0b013e31828132cb

[93] Newman KL , Chedid VG, Boden EK. A systematic review of inflammatory bowel disease epidemiology and health outcomes in sexual and gender minority individuals. Gastroenterology. 2023;164(6):866-871. 37087155 10.1053/j.gastro.2022.11.048 PMC11268438

[94] Boyd T , Friedman S. Challenges and opportunities for advancing research and improving care for sexual and gender minorities with inflammatory bowel disease. Inflamm Bowel Dis. 2023;29(4):672-674. 36308303 10.1093/ibd/izac229

[95] Mansoor E , Martin SA, Perez A, et al Epidemiology of inflammatory bowel disease in men with high-risk homosexual activity. Gut. 2022;72(8):1624-1625. 36170381 10.1136/gutjnl-2022-328218 PMC11866980

[96] Lim FA , Brown DV, Jr, Justin Kim SM. CE: addressing health care disparities in the lesbian, gay, bisexual, and transgender population: a review of best practices. Am J Nurs. 2014;114(6):24-34. https://journals.lww.com/ajnonline/fulltext/2014/06000/ce__addressing_health_care_disparities_in_the.21.aspx 10.1097/01.NAJ.0000450423.89759.36 24826970

[97] Doshi-Velez F , Avillach P, Palmer N, et al Prevalence of inflammatory bowel disease among patients with autism spectrum disorders. Inflamm Bowel Dis. 2015;21(10):2281-2288. 26218138 10.1097/MIB.0000000000000502

[98] Collins PH. Intersectionality’s definitional dilemmas. Annu Rev Sociol. 2015;41(1):1-20.

[99] Dos Santos Marques IC , Theiss LM, Baker SJ, et al Low health literacy exists in the inflammatory bowel disease (IBD) population and is disproportionately prevalent in older African Americans. Crohn’s & Colitis 360. 2020;2(4):otaa076. 10.1093/crocol/otaa076 PMC7802758 33442671

[100] Ahmed S , Singh H, Newton P, Ojo O, Dibley L. N18 Exploring the experiences of UK-dwelling Bangladeshi native language-speaking patients with inflammatory bowel disease: preliminary findings from a qualitative study. J Crohns Colitis. 2023;17(Supplement_1):i1050-i1050.

[101] Mukherjee S , Beresford B, Sebastian S, And AK. Living with Inflammatory Bowel Disease: The Experiences of Adults of South Asian Origin. University of York; 2015.

[102] Flanagin A , Frey T, Christiansen SL; AMA Manual of Style Committee. AMA Manual of Style Committee. Updated guidance on the reporting of race and ethnicity in medical and science journals. JAMA. 2021;326(7):621-627. 34402850 10.1001/jama.2021.13304

[103] Clayton JA , Tannenbaum C. Reporting sex, gender, or both in clinical research? JAMA. 2016;316(18):1863-1864. 27802482 10.1001/jama.2016.16405

