# Supplementary material for: Inequalities in Healthcare Access, Experience and Outcomes in Adults With Inflammatory Bowel Disease: A Scoping Review
Source: Inflamm Bowel Dis. 2024 Apr 11;30(12):2486–99. doi: 10.1093/ibd/izae077 (PMC11630313; doi:10.1093/ibd/izae077)
Supplement: izae077_suppl_Supplementary_Material_S1 [file izae077_suppl_supplementary_material_s1.docx]

*Table 2. Summary of inequalities of IBD access, experience and outcomes*

| **Author and date** | **Study objectives** | **Inequity in IBD healthcare access** | **Inequity in IBD healthcare experience** | **Inequity in outcomes of IBD healthcare** |
| --- | --- | --- | --- | --- |
| Nguyen et al., (2010) | To investigate inequalities in access to specialist care and IBD medicines between “Black and White racial groups”. | Lower use of steroid, immunomodulator, and infliximab in Black patients. Black patients faced more difficulty in scheduling appointments with gastroenterologists & had lower use of specialist care compared to White patients with IBD. | Black patients experienced differences in arranging specialist referrals and had more concerns over healthcare costs. | Black patients were more likely to visit the emergency department (ED) for their disease. Visits to the ED department decreased as income quartiles increased, independent of ethnicity. No difference in the no. of hospitalisations between groups. |
| Mukherjee et al. (2021) | To explore experiences of South Asian population in uk including support received from gastroenterology services | Reported good access to the IBD team outside of scheduled appointments and valued information provided. Access to dietary advice tailored to the SA advice is needed. Two participants experienced lack of access to translation services. | Patients felt appointments focused on medical management and not wider psychosocial elements.Some reported experiences in deficiencies in culturally competent care such as an understanding of what constitutes a 'good' wife or mother and the need to find treatments that enabled them to fulfil these roles. | SA population were more likely to conceal disease, dietary habits might worsen the symptoms, impact of IBD on gender roles (women experience more difficulties in handling disease), increased pressure to visit a faith healer. |
| Ore et al. (2022) | To evaluate racial disparities in postoperative outcomes between “Black, Asian and Hispanic Whites” | Not reported | Not reported | Black patients are at increased risk of morbidity after elective surgery for both UC and CD. Increased stoma creation rates for "Hispanic" and "Asian" patients |
| Dos Santos Marques et al. (2020) | To evaluate racial disparities in surgical outcomes amongst “Blacks, Asians and Hispanics” compared to “White” patients | Not reported | Not reported | “Asian” patients had the longest hospital length of stay (LOS), “Hispanic” patients had the highest rate of readmission, “Black” patients highest complication rates amongst groups |
| Barnes et al. (2021) | To compare racial disparities in medication use for IBD amongst "Blacks" compared to "Whites" with equal access to healthcare | No disparities in accessing medications between the two groups. Black patients had slightly higher combination therapy access and initiated therapies more quickly after surgery that White patients which is reflective of the more complicated disease phenotype that persists in Black patients with IBD | Not reported | No disparities in the use of IBD-specific medications among Black patients with UC or CD when compared with use among White patients with similar socioeconomic background and access |
| Dos Santos Marques et al. (2022) | To evaluate racial disparities in surgical outcomes amongst “Blacks, Asians and Hispanics” compared to “White” patients |  | Not reported | “Asian” patients had the longest LOS, “Hispanic” patients having the highest rate of readmission, “Black” patients highest complication rates |
| Montgomery et al. (2018) | To investigate racial disparity in mortality and morbidity within 30 days of surgical intervention for IBD | Not reported | Not reported | After adjustment for confounders, the study reported that Black patients were at a higher risk of mortality and morbidity within 30 days of post operative procedure. |
| Walker et al. (2018) | To explore differences in the rate of hospitalisation between “African” and “Caucasian Americans” as well as between different socioeconomic class | Not reported | Not reported | “African Americans” and People living at below the poverty level are more likely to be hospitalised for a CD-related event than “Caucasian American”s or people living above the poverty level, adjusting for other factors |
| Borren et al. (2017) | To explore the relationship between distance from specialist care and outcome of disease in IBD | Reduced access to specialist care for people living further away from tertiary hospital which resulted in poorer health outcomes (described in outcomes section) | Not reported | Distance from the referral hospital was associated with an increased requirement for surgery, need for immunomodulator, and biological therapy. |
| Rubin et al. (2017) | To assess IBD patients' ability to access and use care, as well as the timeliness, affordability, and financial stressors related to care. | Many patients reported delays in accessing medical care due to cost concerns | Not reported | A high percentage of patients couldnt acquire insurance coverage. Patients with previous insurance were also worried about additional costs for specialist care and surgery if required in future. Many patients forego treatment to save money on insurance and this was linked to their socioeconomic status, education, income and age.  The risk for ED visit was higher in younger patients (>40), females, patients with lower income and “non White patients”. |
| Benchimol et al. (2016) | To explore health service utilisation and outcomes in immigrant population compared to non-immigrants with IBD | Immigrants had greater outpatient and specialty care compared to non-immigrants. Immigrants were more likely to visit a gastroenterologist (suggesting appropriate use and response of health system) | Not reported | No differences found in time to diagnosis between both groups. Immigrants were less likely to have intestinal resection within 5 years of diagnosis. Both groups had similar hospitalisation and ED visit rates. |
| Govani et al. (2016) | To investigate steroid use and its complication in elderly IBD patients compared to young | Not reported | Not reported | Th elderly population (>65) were less likely to be prescribed steroids. When they were prescribed, the exposure time was greater compared to younger age groups. They were also at a lower risk of being prescribed steroid sparing drugs. |
| Gunnells et al. (2016) | To investigate disparity in readmission post colorectal surgery in black IBD patients compared to white patients | Not reported | Not reported | Black patients were more likely to have readmission post surgery than white patients even after adjusting for confounders |
| Sewell et al. (2010) | To understanding healthcare utilisation of inpatient services by “minorities” in the US | Increase in the utilisation of inpatient services by “minorities” in the United States | Not reported | The hospital discharges for “Asian” and “Black” IBD patients increased between 1994 and 2006 while stayed the same for “native Americans” |
| Nguyen et al. (2009) | To evaluate disparities in the utilisation of parenteral nutrition (PN) among IBD patients belonging to different racial groups | Proportion of "African Americans" receiving PN was lower than "Whites". "No difference in Hispanics" and "non Hispanic Whites". Use of PN varied significantly by geographic location (e.g., Northeast vs Midwest) | Not reported | Delay was observed in the initiation of PN in “African American” and “Hispanics”. Catheter infections were more common in “Hispanics”. |
| Li et al. (2008) | To investigate differences in the standardised incidence ratios (SIR) for admission between people of different literacy level and occupation | Not reported | Not reported | Decrease SIR`s were noted for people with educational level >12. Increased SIR`S were noted for men with exposure to chemicals in their occupation. Although overall a minor effect shown. |
| Herman et al. (2023) | To investigate disparities in surgical outcomes following the creation of the Ileal pouch-anal anastomosis in “minority ethnicities” | Not reported | Not reported | No disparity in surgical outcomes post IPAA creation was noted between minorities and the “White” population. “African American” patients were at a higher risk of developing postoperative bleeding than their “White counterparts”. |
| Straus et al. (2000) | To investigate disparities in disease severity, course, quality of life and access to care between “Black” and “White” Americans with Crohn's disease | Access to medication was similar. Black patients experienced more difficulty in arranging appointments and travelling to healthcare institutions | Black patients experienced delay in appointments due to more difficulty in affording healthcare and experienced unreasonable delays at their appointments | Quality of life was much lower in Black patients and more work days lost to disease. Similar numbers of surgeries and hospitalisations shown between the two groups. |
| Benchimol et al. (2018) | To investigate differences in access to and use of healthcare services between rural and urban population with IBD | Outpatient physician visit rate was similar in rural and urban patients. Rural patients had fewer IBD-specific gastroenterologist visits and a smaller proportion of their IBD-specific care was provided by gastroenterologists | Not reported | Surgical risk was similar in both groups. Compared to urban patients, rural patients had greater rates of hospitalisation and ED visits. |
| Nahon et al. (2009) | To compare the disease severity of Crohns between socioeconomically deprived and non deprived population | Not reported | Not reported | Socioeconomic deprivation was not linked to more severe disease. Increase in rate of hospitalisation of deprived patients. There was a higher rate of IBD linked surgeries in non deprived patients |
| Frieder et al. (2022) | To investigate racial disparity in outcomes in individuals receiving segmental colectomy for IBD | Not reported | Not reported | “African Americans” who underwent segmental colectomy for Crohn's disease or diverticular disease had a greater rate of postoperative complications and a longer hospital stay |
| McKenna et al. (2019) | To assess the impact of race on hospital presentation, operation choice and post op morbidity | Not reported | Not reported | Compared to “non-Hispanic White” patients, “Black” and “Hispanic” patients received non-elective surgery more frequently and had higher postoperative morbidity. However, there were no racial discrepancies in the decision to undertake an initially restorative procedure or the use of laparoscopy. |
| Yarur et al. (2014) | To examine the rate of postoperative problems among “Hispanic” and “non-Hispanic” patients who had equal access to health care. | Not reported | Not reported | No differences in surgical outcomes were found. |
| Arsoniadis et al. (2017) | To investigate the impact of “African American race” on CD surgery outcomes | Not reported | Not reported | Overall, “African American” patients had a much greater risk for complications than “non-African American” individuals. The most serious complications were postoperative sepsis and surgical site infection. |
| Anyane-Yeboa et al. (2018) | To compare the postoperative recurrence rates between “African Americans” and “Caucasians” | Not reported | Not reported | The “African American” race was shown to be substantially linked with clinical recurrence, no difference in endoscopic recurrence |
| Cohen-Mekelburg et al. (2019) | To determine the frequency of delay and risk factors related with a delay in the commencement of preventive post-surgical biologic treatment in high-risk patients | Not reported | Not reported | Delay in post-surgical therapy was observed among Medicare, Medicaid, and uninsured patients |
| Sobotka et al. (2018) | To determine the risk score integrating ethnicity for 30-day readmission in UC patients who have had a colectomy or a proctectomy. | Not reported | Not reported | “Hispanic” UC patients have a greater risk of postoperative problems and are more likely to be readmitted compared to “White and African Americans”. |
| Olaiya et al. (2020) | To investigate temporal patterns and treatment outcomes after TAC in hospitalised UC patients who were “White”, “Black and “Hispanic” | Not reported | Not reported | “Black” and “Hispanic” people had a higher risk of morbidity. “Black and Hispanic” patients had a higher risk of dying in elective instances. Morbidity Odds Ratio rose in rural and low-volume hospitals. Medicare and Medicaid increased odds of morbidity and death. |
| Jackson et al. (2008) | To compare the course and nature of CD in “African-Americans” and “Whites”. | “Whites” were more likely to seek CD treatment in a hospital setting | Not reported | “African-American” individuals had a higher prevalence of colonic disease and Perirectal fistulae. |
| Barnes et al. (2018) | To investigate the association between race and clinical outcome of IBD | Not reported | Not reported | “African Americans” were more likely to develop complications. A similar rate of hospitalisations was found between “White” and “Black” patients. |
| Nguyen et al. (2015) | To investigate the association between age and healthcare utilisation in IBD | The elderly were less likely than young adults to have any IBD-specific gastroenterology visit in the first year after diagnosis and less frequently received continuous gastroenterology care | Not reported | Overall, healthcare utilisation is lower amongst elderly than among the young. |
| Alexakis et al. (2015) | To explore the Challenges faced by young adults from Black, Asian and ethnicity minority community with IBD | Participants reported an absence of culturally competent services that were responsive to the families' communication needs. | Limited capacity of parents to provide assistance due to the lack of culturally competent programmes that were sensitive to the communication requirements of the family, | Not reported |
| Li et al. (2014) | To investigate ethnic disparities between healthcare utilisation and outcomes between UC patients in an integrated healthcare organisation | Not reported | Not reported | Overall health-care utilisation patterns and clinical outcomes were similar across races and ethnicity |
| Stamatiou et al. (2022) | To investigate the impact of ethnicity and socioeconomic background on IBD outcomes | Not reported | Not reported | Patients with CD from “ethnic minority backgrounds” (does not specify) and higher deprivation had increased need for intra-abdominal surgery and surgical complications within 5 years of initial presentation. No such association was found for patients with UC. |
| Nguyen et al. (2007) | To investigate impact of ethnicity, income and type of insurance on surgical outcomes | Not reported | Not reported | “African Americans”, “Hispanics”, and “Asians/Pacific Islanders' ' hospitalised for CD have lower rates of surgical resection.People with private insurance had lower risk of surgery. People living in high income neighbourhoods had lower mortality. |
| Nguyen et al. (2006) | To investigate impact of ethnicity, income and type of insurance on surgical outcomes | Not reported | Not reported | Colectomy rates were lower in “Blacks”. Medicaid patients had higher mortality rates compared to those who had private insurance. “African Americans” had hospital LOS. |
| Galooisan et al. (2020) | To evaluate ethnic disparities in IBD related hospitalisation outcomes | Not reported | Not reported | “Hispanic” patients with CD had longer LOS compared to all groups. “Hispanic” and “African American” patients with UC had higher in-hospital mortality trends compared to “non-Hispanic Whites”. |
| Kuenzig et al. (2020) | To assess the variation in access to care in patients diagnosed with IBD 65 yrs and above | Variation found in the odds of over 65s ever seeing a gastroenterologist or having a gastroenterologist as their primary provider. Patients who were in networks with fewer gastroenterologists were less likely to see one and to have a gastroenterologist as their primary provider. | Not reported | No variation in ED visits, hospitalisations or resection for CD was found. |
| Odufalu et al. (2023) | To identify healthcare disparities in relation to social determinants of health and emotional impacts related to disease management and patient experience | Low income patients were less likely to access UC education programmes or peer mentoring. Patients younger than 50 yrs vs 50 and older were less likely to have accessed an IBD centre within the past 12 months. Males were less likely to access a gastroenterologist. | Not reported | Not reported |
| Richard et al. (2020) | Explored how people living in rural New Zealand engaged with healthcare providers | Rural IBD patients experienced challenges in managing their IBD due to access to specialist care. Patients experienced delayed referrals and expressed concerns about disparities in specialist access compared with urban areas. | Living far from the main health centre left some participants feeling like they were ‘falling through the cracks’ and ‘forgotten about’ | Not reported |
| Rohatinsky et al. (2021) | Examined healthcare utilisation and access to care for rural adults with IBD. | Lack of local services such as outpatient clinics, hospitals, laboratory testing, infusion clinics, and pharmacies meant individuals with IBD frequently had to travel to access care. | Communication with HCPs was challenging due to distance to access care. Patients felt rural HCPs had a lack of IBD related knowledge. Participants described frequently experiencing gaps in care. | Not reported |
| Greenstein et al. (2013) | Assessed if UC patients with Medicaid who presented for subtotal colectomy would have reduced access in comparison with a similar population with private insurance | Not reported | Not reported | Patients with private insurance were more likely to undergo the laparoscopic STC than those with standard Medicaid insurance. |
| Dibley et al. (2014) | Aimed to identify the social and psychological aspects of IBD in the gay and lesbian population | Not reported | Gay and lesbian patients felt as though they were treated differently when receiving healthcare including not having their partner acknowledged during consultations or understanding their emotional support needed during encounters. | Not reported |
| Flasar et al. (2008) | Assessed differences in biologics assessed between “African Americans” and “White Caucasians” | Trends found toward lower odds of treatment with IFX or either 6-MP/AZA or IFX in African Americans when compared with Caucasians | Not reported | Not reported |
| Bhurwal et al. (2022) | Described racial and geographic disparities in colectomy rates among patients hospitalised with UC after anti-TNF therapy was introduced | Compared to “Black” and “Hispanic” patients, “White” IBD patients had greater rates of colectomy. Geographic differences in colectomy rates also shown, patients admitted to urban, larger, and teaching hospitals had higher rates of colectomy. | Not reported | Mean hospital LOS did not differ between ethnic groups. Having Medicare compared to private insurance was also associated with inferior colectomy rates. |
| Nordenvall et al. (2021) | Explored the association between income and restorative surgery after colectomy. | Not reported | Not reported | The chance of having restorative surgery after a colectomy (greater access to) was higher for IBD patients on a high income. No associations found between socioeconomic factors (eg., income, education, employment) and the risk of success or failure of the surgery. |
| Farrukh & Mayberry (2015) | Investigated if South Asian IBD patients with CD received the same access to biological therapy than English patients | Based on the proportions of who should have received Infliximab treatment, the number of South Asian patients receiving biological treatment was significantly less than the European patients | Not reported | Not reported |
| Farrukh & Mayberry (2016) | Investigated whether South Asians experienced the same quality of care as English patients with ulcerative colitis | English patients were more likely to be reviewed by a consultant than South Asians. English patients had significantly more investigations covering a wider range of modalities than did South Asian patients when emergency investigations were required. | Not reported | South Asian patient with ulcerative colitis was 2.6 times more likely to be discharged from hospital follow-up than a “European” patient |
| Farrukh & Mayberry (2022) | Investigated disparities in optimal surgical care between South Asian and British patients with IBD | In patients with a severe flare of UC and when surgery is warranted, the nature of the procedures offered was the same in the White British and South Asian communities | Not reported | Not reported |
| Axelrad et al. (2019) | Understood healthcare use in IBD patients based on health insurance status used as a proxy for low-socioeconomic status (Medicaid) | Not reported | Not reported | Patients with Medicaid cover compared to privately insured patients had higher rates of hospital admissions, ED visits, and requiring steroid treatment. |
| Lin & Sewell (2013) | Examined disparities in “minority ethnicity” and socio economic status for ambulatory patients with IBD | No differences or evidence of disparity between ethnicity (“Hispanic, Black and Asian”) and socioeconomic status in accessing biologics when admitted to emergency care | Not reported | Not reported |
