# Supplementary material for: Inequalities in Healthcare Access, Experience and Outcomes in Adults With Inflammatory Bowel Disease: A Scoping Review
Source: Inflamm Bowel Dis. 2024 Apr 11;30(12):2486–99. doi: 10.1093/ibd/izae077 (PMC11630313; doi:10.1093/ibd/izae077)
Supplement: izae077_suppl_Supplementary_Material_S4 [file izae077_suppl_supplementary_material_s4.docx]

**EMBASE Search**

1. Inflammatory bowel disease/
2. Crohn disease/
3. Ulcerative colitis/
4. Ibd.mp.
5. Crohns.mp.
6. Ulcerative colitis.mp.

7 OR 1-6

1. Healthcare disparity/
2. Healthcare access/
3. Health inequalities.mp.
4. Healthcare experience/
5. Patient experience/
6. experience/
7. Health care delivery/
8. Health Services Accessibility/

16 Or 8-15

1. Discrimination.mp.
2. Equity.mp.
3. socioeconomic disparities in health.mp.
4. Minority group/
5. Minority health/
6. poverty/
7. (Black or African American).mp.
8. aged/
9. Educational status/
10. ethnicity/
11. EthniCITY.mp.
12. Race relation/
13. Racial disparity/
14. Racial background/
15. Social class/
16. Social isolation/
17. Social problem/
18. Social status/
19. Gender equity/
20. Vulnerable population/
21. Women.mp.
22. Social class.mp.
23. Rural healthcare/
24. Rural health/
25. Income.mp.
26. Ethnic group/
27. Cultural diversity/
28. Barriers.mp.
29. socioeconomics/
30. Elderly.mp.
31. Distance to healthcare.mp.
32. 'lgbtqia+ people'/exp
33. 'sexual and gender minority'/exp
34. 'men who have sex with men'/exp
35. 'men who have sex with men and women'/exp
36. 'women who have sex with women'/exp
37. 'women who have sex with women and men'/exp
38. 'asexual people'/exp
39. 'intersex'/exp
40. 'lgbt people'/exp
41. 'bisexual female'/exp
42. 'bisexual male'/exp
43. 'homosexual female'/exp
44. 'homosexual male'/exp
45. 'transgender'/exp
46. Sexual minority/

63 OR 17-62

7 AND 16 AND 63

Limit 2000-current

**Medline via Ovid**

1. Inflammatory bowel disease/
2. Crohn disease/
3. Ulcerative colitis/
4. Ibd.mp.
5. Crohns.mp.
6. Ulcerative colitis.mp.
7. OR 1-6
8. Healthcare disparity/
9. Healthcare access/
10. Health inequalities.mp.
11. Healthcare experience/
12. Patient experience/
13. experience/
14. Health care delivery/
15. Health Services Accessibility/
16. OR 8-15
17. Discrimination.mp.
18. Equity.mp.
19. socioeconomic disparities in health.mp.
20. Minority group/
21. Minority health/
22. poverty/
23. (Black or African American).mp.
24. aged/
25. Educational status/
26. ethnicity/
27. EthniCITY.mp.
28. Race relation/
29. Racial disparity/
30. Racial background/
31. Social class/
32. Social isolation/
33. Social problem/
34. Social status/
35. Gender equity/
36. Vulnerable population/
37. Women.mp.
38. Social class.mp.
39. Rural healthcare/
40. Rural health/
41. Income.mp.
42. Ethnic group/
43. Cultural diversity/
44. Barriers.mp.
45. socioeconomics/
46. Elderly.mp.
47. Distance to healthcare.mp.
48. Sexual minority/
49. asexual/
50. intersex/
51. transgender/
52. lesbian/
53. gay/
54. homesexual/
55. OR 17-55
56. 7 AND 16 AND 55

Limit 2000-current

**CINAHL Search**

inflammatory bowel disease or ibd or ulcerative colitis or crohn's disease, TI

AND healthcare access or health access or access to healthcare or health services accessibility OR healthcare experience OR healthcare services or healthcare access or healthcare utilization AND minority groups or minority health OR health inequalities or health inequities or social determinants of health or health disparities OR ethnicity or race or culture or minority or minorities or ethnic backgrounds OR deprivation or poverty or low income or socio-economic OR gender inequality or sexism or gender discrimination or gender bias OR lgbtq or lesbian or gay or homosexual or bisexual or transgender or homosexual or queer or sexual minority OR rural areas or rural communities

Limits: 2000-2023, all adult, english langauge
